# Supplementary material for: A real-time, multi-animal model for automatic face detection and identification of freely moving common marmosets based on YOLOv8 algorithms
Source: eLife. 2026 Sep 29;15:RP110932. doi: 10.7554/eLife.110932 (PMC13623377; doi:10.7554/eLife.110932)
Supplement: Peer review history

# Editor report: eLife Assessment

**DOI:** 10.7554/eLife.110932.3.sa0

**Authors:** Arun SP

**Affiliations:** Indian Institute of Science Bangalore India

**Keywords:** Solid, Valuable

This **valuable** study presents a real-time system for identifying multiple unrestrained marmosets in a home cage setting using a combination of facial features and color-coded beads. While there is **solid** evidence that the system has a precision comparable to human experimenters in the tested scenarios, there is limited evidence that this would generalize to unconstrained multi-animal environments

# Referee report: Reviewer #1 (Public review)

**DOI:** 10.7554/eLife.110932.3.sa1

The manuscript by Yang, Wang, and Cléry presents a pipeline for real-time identification of common marmosets in a laboratory setting. Models were trained and evaluated on data derived from a family of three closely related adults and a set of juvenile twins. Freely moving animals entered an enclosed space fixed to the housing cage door, which permitted the entry of individual animals for data acquisition. Utilizing YOLOv8-nano, identification was improved through the introduction of uniquely colored collar beads. Analyses of facial similarity showed close morphological relatedness amongst individuals and highlighted the need for highly discriminative classification. The authors demonstrate that combining facial detection with visual markers enables adequate identity assignment under controlled laboratory conditions with minimal cross-individual misclassification.

The main strengths are that the proposed pipeline offers a solution for real-time identity tracking in common marmosets. Its lightweight design enables deployment across a wide range of hardware configurations. Furthermore, if similar strategies are employed, this methodology is likely adaptable for other species with minimal modification. Additionally, evaluation of closely related individuals provides a necessary stress test for the discrimination of facial identity tracking. However, the main weakness is the pipeline's reliance on controlled animal isolation and small visual markers, which raises questions about the approach's generalizability to unconstrained multi-animal environments. The authors justify the use of beads, but the dependency of facial recognition on the beads needs to be described more clearly, as it is unclear how independent facial recognition performance truly was. The overall utility of this approach therefore remains to be seen.

# Referee report: Reviewer #2 (Public review)

**DOI:** 10.7554/eLife.110932.3.sa2

Summary:

In this study, Yang et al. develop a real-time system for automatic face detection and identification of multiple unrestrained common marmosets in a home cage setting.

Strengths:

The study aims to address an unmet need in behavioral neuroscience: the ability to non-invasively identify animals is crucial to the automated and rigorous study of neural behaviors; this is especially true for common marmosets, which are rapidly becoming a model system of choice for the study of complex social cognition. By using a YOLOv8 backbone, the study achieves human level performance, both in terms of precision and recall of the trained models.

Weaknesses:

The robustness of the system is not clear from the limited datasets presented.

Comments on revised version.

The authors have adequately addressed my comments from the previous round, and I have no further comments

# Referee report: Reviewer #3 (Public review)

**DOI:** 10.7554/eLife.110932.3.sa3

Summary:

In the revised manuscript, the authors provide additional details and evidence regarding the robustness and utility of their method.

Strengths:

(1) The authors provide a very precise automatic identification of marmosets in their home cage, to levels comparable to animal health professional.

(2) This method is robust across lightning, camera angles etc but importantly is able to identify marmosets in naturalistic conditions, which can be of tremendous value to neuroscientists and to ecological or behavioral studies.

(3) Easy to use and implement, requiring minimal settings. Phone videos can even be used.

Weaknesses:

While the manuscript improved tremendously from the previous version, given the nature of the paper, it is still a strenuous read.

Comments on revised version.

The authors did a good job of addressing my previous concerns and I don't have more comments.

# Author comment: Author response

**DOI:** 10.7554/eLife.110932.3.sa4

**Authors:** Jiayue Yang, James Wang, Justine Cléry

**Affiliations:** McGill University Montreal Canada; McGill University Montreal Canada; McGill University Montreal Canada

The following is the authors’ response to the original reviews.

**Public Reviews:**

**Reviewer #1 (Public review):**

Summary:

The manuscript by Yang, Wang, and Cléry presents a lightweight pipeline for real-time identification of common marmosets in a laboratory setting. Models were trained and evaluated on data derived from a family of three closely related adults and a set of juvenile twins. Freely moving animals entered an enclosed space fixed to the housing cage door, which permitted the entry of individual animals for data acquisition. Utilizing YOLOv8-nano, identification was improved through the introduction of uniquely colored collar beads. Analyses of facial similarity showed close morphological relatedness amongst individuals and highlighted the need for highly discriminative classification. Overall, the authors offer a framework for identity tracking that prioritizes real-time inference. The authors demonstrate that combining facial detection with visual markers enables adequate identity assignment under controlled laboratory conditions with minimal cross-individual misclassification.

Strengths:

(1) The proposed pipeline offers a solution for real-time identity tracking in common marmosets. Its lightweight design enables deployment across a wide range of hardware configurations. Furthermore, if similar strategies are employed, this methodology is likely adaptable for other species with minimal modification.

(2) Evaluation of closely related individuals provides a necessary stress test for the discrimination of facial identity tracking.

Weaknesses:

(1) The pipeline's reliance on controlled animal isolation and small visual markers raises questions about the approach's generalizability to unconstrained multi-animal environments. The provided confusion matrices (Figures 6-8) indicate that the most common misclassifications are background-related, possibly suggesting that detection specificity is the primary source of error. All things considered, these findings raise concerns about performance in its use in socially dynamic and visually complex environments.

Thank you for the comment. The background column of the confusion matrix can be explained by several occasions: (a) the model detects an object where there is no object, (b) there is more than one prediction label for the same object, or (c) an object appeared in the image but not manually labeled, however the program was able to detect that object. The value of the background column does not necessarily mean that the detection is incorrect, as the precision score for the detection labels are good. We have rephrased the relevant sections for clarification to include the sources of the increased value in background columns in confusion matrices, as follows:

“The background class of the confusion matrix showed frequently predictions as marmoset faces and collar beads for the training (Figure 6A) and validation set (Figure 6B). However, it does not necessarily indicate incorrect predictions or misclassifications. Instead, these values were mostly explained by multiple detections of the same object class. For instance, additional marmoset faces were predicted when multiple animals were present within a single video frame. The long collar structure or motion blur of the marmosets could also cause multiple detections of beads that belong to the same collar. This also corresponded to the high precision and recall scores observed across prediction classes (Figure 5D), suggesting that the increased background false positives were mainly related to the object-count discrepancies, instead of poor detection performance.”

Prediction misclassification is one source of the background false positive. The misclassification could not be avoided in automatic prediction algorithm, but we included the manual filtering and majority-voting during our real-time classification to reduce this effect. Multiple detection of the same class may also be considered as the background, since only one object may be labelled in the ground truth, such as multiple collar beads or automatic face extraction. In addition, blurry objects were not labeled manually during training but can be detected during prediction, which also resulted in background false positive. It was clarified in the main text as follows:

“The normalized confusion matrices showed high accuracy and consistency of most marmoset faces and collars detection in training (Figure 8A) and validation (Figure 8B) tests, with some exceptions. Particularly, the background was frequently identified as the collar of Young2 marmoset. This elevated background score was likely contributed by the multi-color design of the Young2 marmoset collar, making it more difficult to distinguish compared to collars with a single bead color. In this occasion, if one bead is occluded, blurred, or outside the field of view, the other visible collar bead could affect the prediction and lead to an incorrect identification from the ground truth.”

(2) The manuscript claims performance comparable to that of human experimenters but provides no explicit evidence to support these claims. While it is plausible that human experimenters may be less accurate in facial recognition tasks involving closely related marmosets, the authors don't provide evidence. Moreover, while that might be the case, the color-coded beads provide a salient identity cue for the model, which complicates the interpretation of this comparison grounded in facial recognition.

Thank you for pointing out this concern. The aim of the facial recognition tool is to collect data from marmosets without having experimenters to check the identity continuously. The program is not aimed at outperforming the experimenters’ role but avoid having constant human intervention that can disrupt a more ecological in cage data collection. It is also essential for having more flexibility to collect data in case a specific experimenter is not here and thus to not disrupt the project. Human experimenters have extensive experience closely working with marmosets, having the unique collar beads associated to each marmosets allows human experimenters to hardly make mistakes identifying marmosets and to do it quickly. We collected identification accuracy of human experimenters by presenting 10 clips of the five marmosets involved in the manuscript (2 clips per marmosets), with 2 random clips repeated twice. The results were plotted by each experimenter. The identification accuracy of the experimenters correlates with the time spent with the animals, as the animal health technicians (responsible for daily health check and husbandry) achieved 95.83% average accuracy in identifying the marmosets. We clarified those points in the main text as follows:

“Its automated pipeline substantially reduces the time and work required for traditional manual identity labeling, while maintaining an expert-level human performance and reproducibility across experimenters (95.83% average accuracy for animal health technicians, responsible for daily health check and husbandry while lab experiments ranges between 25 to 80% of accuracy depending on the amount of time spent with each animal, Supplementary figure 1). The tool’s advantages are particularly efficient for large datasets and longitudinal studies, where manual identity labeling becomes difficult, as variability and errors increase along with dataset size and experimenter number.”

We filtered the prediction of the collars, and the identification result solely based on the faces for the 2 young marmosets was correct. The prediction results were plotted on Video 7, Video 7—video supplement 1, and Video 7—video supplement 2 and added to the Results section as follows:

“We tested the prediction performance without collar and its longitudinal application, using the face-only prediction on the young marmosets at 11 months and 16 months (Video 7, Video 7—video supplement 1, Video 7—video supplement 2). The face classifier correctly identified the young twin marmosets solely based on their facial features, indicating that facial identity classification was performed independently of collar information and that the collar beads acted only as an auxiliary confirmation rather than the main classifier of the system (Video 7).”

Explanation for classification of marmoset faces and collar beads in the Discussion section:

“Facial features serve as the main and intrinsic biometric identifier for each marmoset, providing a unique source of individual recognition. Since collar-based confirmation could be affected by visibility limitations, we implemented the uniquely color-coded bead collars as an auxiliary cue to provide additional confirmation in identity prediction. For example, this issue can be caused by identical or similar bead colors between individuals (Video 5, 6) and beads that are occluded by fur (Video 3 - 6). In addition, collar beads may change over time or not be worn by all animals.”.

“With one separated model trained per family unit, our system can utilize distinct collar colors as an additional identifier when available, while facial features performed as the main biometric marker. Even though multiple marmosets with visually similar faces may present close to the camera, the additional collar information can improve confidence in identity prediction without replacing facial recognition as the primary mechanism of identification (Video 7).”

**Reviewer #2 (Public review):**

Summary:

In this study, Yang et al. develop a real-time system for automatic face detection and identification of multiple unrestrained common marmosets in a home cage setting.

Strengths:

The study aims to address an unmet need in behavioral neuroscience: the ability to non-invasively identify animals is crucial to the automated and rigorous study of neural behaviors; this is especially true for common marmosets, which are rapidly becoming a model system of choice for the study of complex social cognition. By using a YOLOv8 backbone, the study achieve human level performance, both in terms of precision and recall of the trained models.

Weaknesses:

The robustness of the system is not clear from the limited datasets presented. The use of color-coded beads undercuts the study's premise that the system achieves truly non-invasive tracking. Although the system achieves good performance in face detection, it does not perform as well for classification using faces alone (especially when the faces are similar, as in twin animals). Here, too, the color-coded beads play a key role in identity discrimination. The stated goals of the study and the actual results presented are therefore at odds.

Thank you for the comment. First, we would like to clarify the role of the collar beads in our system. Compared to the faces, a unique identity marker, the collar beads were not used as the main identity classifier but rather as an external visual marker. The color-coded bead was not used solely for the purpose of marmoset video classification; it was also used as an additional source of identification for one marmoset. As the marmosets usually move very fast inside the cage, it is mainly used as a visual marker for experimenters to recognize them in a distance in a short time.

The mislabelling is more frequent with the young twins not only due to their face similarity, but also due to the limited number of images being used for the model training, as discussed in the paragraph #4 of the Discussion section. Collar beads are small and less frequently detected by the camera, since it could be occluded by the marmoset fur. In addition, it was invisible to the camera if the marmoset turned sideways or was far from the camera. Therefore, higher weight was assigned to the beads due to their small size and less frequent detections compared to face labels, such that it was only an element to confirm the identity, instead of the main classifier.

The inclusion of the collar beads doesn’t affect the prediction results of the marmoset faces. The model achieved a good precision/recall score for the identity labeling in the manuscript. In the revision, with the majority-vote strategy, we filtered the detection of all collar beads and showed that the model was able to correctly identify the marmosets solely by their faces. The Results section has been modified as follows:

“We tested the prediction performance without collar and its longitudinal application, using the face-only prediction on the young marmosets at 11 months and 16 months (Video 7, Video 7—video supplement 1, Video 7—video supplement 2). The face classifier correctly identified the young twin marmosets solely based on their facial features, indicating that facial identity classification was performed independently of collar information and that the collar beads acted only as an auxiliary confirmation rather than the main classifier of the system (Video 7).”

Explanation for classification of marmoset faces and collar beads in the Discussion section:

“Facial features serve as the main and intrinsic biometric identifier for each marmoset, providing a unique source of individual recognition. Since collar-based confirmation could be affected by visibility limitations, we implemented the uniquely color-coded bead collars as an auxiliary cue to provide additional confirmation in identity prediction. For example, this issue can be caused by identical or similar bead colors between individuals (Video 5, 6) and beads that are occluded by fur (Video 3 - 6). In addition, collar beads may change over time or not be worn by all animals.”.

“With one separated model trained per family unit, our system can utilize distinct collar colors as an additional identifier when available, while facial features performed as the main biometric marker. Even though multiple marmosets with visually similar faces may present close to the camera, the additional collar information can improve confidence in identity prediction without replacing facial recognition as the primary mechanism of identification (Video 7).”

**Reviewer #3 (Public review):**

Summary:

In this manuscript, Yang et al introduce a new method for automatically identifying marmosets in their home cage using a supervised deep learning method that recognizes the face and colored beads on marmoset collars. The authors show a high precision rate of identifying marmosets to levels comparable to a human experimenter. The method overall seems robust at identifying marmosets at different life stages and different settings; however, given the current form, I'm struggling to see the generalizability and experimental utility of this method.

Strengths:

(1) The authors provide a near-perfect automatic identification of marmosets in their home cage.

(2) This method is robust across lightning, camera angles, etc., making it potentially useful for marmoset (and other NHP) identification outside the housing cage as well

Weaknesses:

(1) Despite the almost perfect precision, in its current form, I'm failing to see how this method can be useful to other labs.

Thank you for your comment. This Tools & Resources paper mainly described the development of the marmoset identification program and methods. Future work will focus on extending the program application on identification from different housing conditions, in combination with various behavioral tasks such as in-cage touchscreen system or manual tasks, and in the wild that precludes handling or isolation of marmosets for collecting behavioral data. The program solely requires a camera, a computing device, and marmosets, as there are no hardware restrictions. In addition, we are currently collaborating with other labs on the marmoset identification from videos taken from other setups. The program achieved effective face extraction from the marmoset in the video, without the need for additional program modifications.

(2) This is a nice methods manuscript, but the authors do not present results to show how their method can be used outside of identifying marmosets inside their home cages in a small field of view.

Thank you for your feedback. The method developed was applied in combination with other touchscreen behavioral tasks, aiming to extract data without human intervention. This approach was discussed in paragraph #6 in the Discussion section. While this manuscript focuses on the methods of close-view face identification when marmosets perform behavioral tasks, the identification and automatic face extraction program could also be applied to marmoset videos taken from a larger view, including phone cameras. Even though the marmosets are still housed in their home cage, the example videos presented the program’s application in a larger field of view. We have added examples of the videos/photos from a different experimental setup to respond to this comment in the Discussion section as follows:

“The motivation for this real-time marmoset identity recognition program was to develop an easy-to-use, generalizable pipeline that could be applied across different marmosets and lab environments, such as using larger field of view or phone cameras (Figure 10).”

(3) Reading the manuscript is strenuous, given its repetitive nature. Consolidating and shortening the results, as well as adding some definitions to the results section, would be helpful.

Thank you for pointing this out and your suggestions. We have rephrased the Results section for simplification to facilitate the understanding of the manuscript.

**Recommendations for the authors:**

**Reviewer #1 (Recommendations for the authors):**

(1) The weight of the color-coded beads was increased to improve identification accuracy. From a brief look at the code provided on GitHub, the weight assigned to the beads seems substantial. This calls into question the need to use facial recognition in the identification strategy. As the code currently stands, facial identity appears to serve primarily as a fallback when bead detection fails to register. To strengthen the methodological justification, the paper would benefit from the authors providing a rationale for choosing this weighting scheme and, if available, supplemental figures showing performance across a range of different weights to demonstrate why that specific value was assigned in the algorithm.

We have added the model prediction results without the collar beads showing that the facial recognition algorithm works even without the collar beads and that those collar beads are not the main classifier. The Methods section has been modified as follows:

“For each detected bounding box, the scripts returned a corresponding label of marmoset face and collar bead color. We assigned the detected collar beads as the corresponding marmoset identity with a higher weight, which improved the detection confidence across frames.”

The Discussion section has been modified as follows:

“Facial features serve as the main and intrinsic biometric identifier for each marmoset, providing a unique source of individual recognition. Since collar-based confirmation could be affected by visibility limitations, we implemented the uniquely color-coded bead collars as an auxiliary cue to provide additional confirmation in identity prediction. For example, this issue can be caused by identical or similar bead colors between individuals (Video 5, 6) and beads that are occluded by fur (Video 3 - 6). In addition, collar beads may change over time or not be worn by all animals.”

“With one separated model trained per family unit, our system can utilize distinct collar colors as an additional identifier when available, while facial features performed as the main biometric marker. Even though multiple marmosets with visually similar faces may present close to the camera, the additional collar information can improve confidence in identity prediction without replacing facial recognition as the primary mechanism of identification (Video 7).”

The weight assigned to the beads in the GitHub page is the highest weight that we would suggest. The actual weight can be customized by the experimenters based on the actual experimental setup. For example, we used the weight of 2 in our real-time version of the marmoset face identification, while marmosets were presented with their corresponding tasks once identified. The GitHub page has been edited to clarify this point.

(2) The overall utility of this approach, other than the real-time detection component, needs more clarification. It is currently unclear why this approach, and in which specific experimental or observational settings, is particularly advantageous compared to existing methods for assigning animal identity.

In addition to the advantages mentioned in paragraph #1 of the Introduction and paragraphs #1-3 in the Discussion, we have added more details in the Discussion section:

“While existing marmoset identification approaches usually utilize visible markers, Radio Frequency Identification (RFID), or observation, the manual works and human interventions involved can impact animal behaviors, especially during their behavioral task performance. The facial identification tool aims to collect data from marmosets without having experimenters to check the identity continuously, instead of outperforming the experimenters’ role.”

(3) Although it appears that performance based on faces and color beads was evaluated separately, this was not clearly presented, leading to confusion about whether face detection performance also benefited from color beads on the animals.

Prediction of the different labels in the same model is independent, so the prediction of color beads is not affecting the prediction results of marmoset faces. Correct identity classification could be achieved without depending on the color beads, as we have filtered out the color beads detection class. The Results section has been modified as follows:

“We tested the prediction performance without collar and its longitudinal application, using the face-only prediction on the young marmosets at 11 months and 16 months (Video 7, Video 7—video supplement 1, Video 7—video supplement 2). The face classifier correctly identified the young twin marmosets solely based on their facial features, indicating that facial identity classification was performed independently of collar information and that the collar beads acted only as an auxiliary confirmation rather than the main classifier of the system (Video 7).”

**Reviewer #2 (Recommendations for the authors):**

(1) I found the paper quite confusing as written. The term "model" is overused and highly conflated: there are the YOLOv8 pre-trained models, the "face classification" model, and the "automatic facial and identity extraction" model. The flowchart in Figure 2A is equally confusing. The mapping from the flowchart to the results is not straightforward, and I needed several passes to grasp it. I would recommend that the authors simplify the terminology and the mapping of the methods to the results.

Thank you for pointing this out! The YOLOv8 pre-trained models, the "face classification" model, and the "automatic facial and identity extraction" model were indeed separately trained object detection models. They all have different weights/parameters but share the same YOLOv8 architecture/backbone. We removed some of the “model” term in the manuscript and replaced them with “classifier/framework/pipeline” to avoid misunderstanding. This information has been clarified in the revised manuscript of the Methods section and Figure 2, which provides an overall clearer explanation of the workflow of the methodology of the program.

(2) It is not clear how robust these results are, given the limited data sets analysed.

We agree that only five marmosets were involved in this manuscript, this unfortunately limited the robustness of the prediction results. Indeed, the limited number of animals that can be used per study has been a main limitation in non-human primate research, as they are very valuable animal models. However, we included approximately 3400 images in the training dataset, which were collected across days. New videos and photos that were captured from different devices were also used in the testing to ensure that the program can be used on new marmosets, different housing cage, and from different recording devices as indicated here:

“The motivation for this real-time marmoset identity recognition program was to develop an easy-to-use, generalizable pipeline that could be applied across different marmosets and lab environments, such as using larger field of view or phone cameras (Figure 10). The pipeline was designed to have no specific hardware requirements and can be implemented for any standard recording device, including any commonly available cameras, primate chair system, and computer-based device.”

(3) There are two paradoxes regarding the stated motives of the study:

(a) If the objective was to truly use non-invasive methods for the identification of animals, then why use the color-coated beads?

As mentioned previously, identity detection can be made without collar beads, still with correct prediction results as indicated here:

“We tested the prediction performance without collar and its longitudinal application, using the face-only prediction on the young marmosets at 11 months and 16 months (Video 7, Video 7—video supplement 1, Video 7—video supplement 2). The face classifier correctly identified the young twin marmosets solely based on their facial features, indicating that facial identity classification was performed independently of collar information and that the collar beads acted only as an auxiliary cue rather than the main classifier of the system (Video 7).”

The color-coated beads are used for easier and quick marmoset identification during daily care, health check, for weekend staff, training or handling.

(b) If the objective was to achieve high identification performance, and the color-coated beads are sufficient for this purpose, then why bother with faces at all?

Collar beads are small compared to the face, and less visible due to fur occlusion and motion blur. Moreover, it is possible that some marmosets do not have collar beads due to their young age or when involved in other procedures such as imaging scans. The collars need to be checked and changed regularly in growing marmosets and it is not always convenient (some marmosets do not support the collar, some can have sensitive skin that would lead to abrasion) thus the need to develop a facial recognition system. Furthermore, marmosets who are from other labs or in the wild might not wear a collar with colored beads, thus face is the main classifier in this model to be more generally applicable. It is highlighted here:

“Facial features serve as the main and intrinsic biometric identifier for each marmoset, providing a unique source of individual recognition. Since collar-based confirmation could be affected by visibility limitations, we implemented the uniquely color-coded bead collars as an auxiliary cue to provide additional confirmation in identity prediction. For example, this issue can be caused by identical or similar bead colors between individuals (Video 5, 6) and beads that are occluded by fur (Video 3 - 6). In addition, collar beads may change over time or not be worn by all animals.”

(4) I was puzzled by the face similarity results in Figure 9. It appears that the face similarity measures were stronger (higher cosine similarity, lower Euclidean distance) for the adult data set compared to the twin data set. If so, why was it more challenging for the system to handle the twin data set?

Face similarity can only be compared within models (therefore within adults and within twins). As this is calculated from different models, the adult face similarity cannot be compared with twins’ face similarity. It has been clarified in the Methods section as follows:

“Statistical tests were performed only within the face classifier of each marmoset family, as embedding spaces may vary in scaling, learned features, and baseline metrics making cross-model comparison of inter-individual face similarity unreliable (Bollegala, 2017).”

And in the Results section as follows: “We performed the statistical tests only on the face classifier for the adult marmoset family, as the twin marmoset model only involved two individuals and thus not valid for within-model statistical analysis (Table 2, 3).”

The twin dataset aims to represent a test for the program utility in new marmosets, especially for testing if the program can still distinguish between the marmosets with similar faces. Thus, the number of twin data collected is less than the adult dataset, as explained by Discussion paragraph #2 “While comparing between the adult and young marmoset datasets, we found that the adult marmosets’ face classifier, trained with a larger number of varied images, showed more reliability and efficiency in marmoset identity recognition.” This explains the challenge the system faces when differentiating the twins, while increasing the training dataset is required to solve this issue.

**Reviewer #3 (Recommendations for the authors):**

Major issues:

(1) My main issue is regarding the utility of this method in scientific experiments. This manuscript is a "methods paper" introducing a face recognition method to identify a single marmoset in their home cage in a very specific and confined field of view. This comprises a limitation on what experiments can be performed using this method. On the contrary, if (a) the authors can show that this method can be used for a bigger field of view, where the social structure/interactions can be studied for neuroethological, cognitive or social studies that will make this method significantly more robust; or (b) design an experiment that can be performed using the current method to show that this method in its current form is sufficient.

(a) Our method worked in larger home cage (larger view) with videos taken inside the cage / outside the cage, with multiple marmosets moving around, while the camera and its fixation are also moving. A new figure (Figure 10) has been added to highlight this wide application:

“The motivation for this real-time marmoset identity recognition program was to develop an easy-to-use, generalizable pipeline that could be applied across different marmosets and lab environments, such as using larger field of view or phone cameras (Figure 10). The pipeline was designed to have no specific hardware requirements and can be implemented for any standard recording device, including any commonly available cameras, primate chair system, and computer-based device.”.

(b) We are currently using this method to collect in-cage touchscreen data with multiple marmosets without the need to isolate such animals to acquire the data, avoiding social separation. The collection of data in nonhuman primates is still a long process, so we wanted to share the facial recognition system first, aligned with our commitment towards open science, to benefit the broader community (we have already been contacted by two labs since the publication of this preprint) while we keep collecting data for the scientific project. We have added the touchscreen application as example in the Discussion section as follows:

“Once trained, the system operates automatically to collect real-time identity and can work to present subject-specific behavioral or cognitive tasks based on the identity of the detected animal, with no work or presence needed on the user’s end. This tool has already been implemented in touchscreen-based marmoset cognitive tasks, including pairwise visual discrimination paradigm.”

(2) The authors claim a longitudinal identification of marmosets, yet I think the data to fully support this are deficient. This might be a result of unclarity of this experiment. How was this experiment done? Was the training done on the 7 months and then applied to the 11 months? Are there more continuous data that track the precision of the identification in time? For example, how does the twin identification evolve in time?

This Tools & Resources paper mainly described the development of the marmoset identification program and methods. Ongoing work in the lab, the main research focus of which is the longitudinal assessment of cognitive functions, either during neurodevelopment or in preclinical ageing model, is benefiting from such algorithms to help identifying the animals to collect in cage behavioral data. As such, we have done some testing in one young cohort. The training of the young marmosets’ identification was done only on the 7-month data, and then we applied the identification program to the videos of the same marmosets when they were 11 months old and 16 months old (for the no-collar results) as indicated as follows in the Methods section:

“Moreover, we evaluated the model performance and its generalization across developmental stages using new videos: (1) from the adult marmosets and (2) from the same young marmosets at 11 months, which were not involved during initial program training”.

And in the Results section as follows: “We tested the prediction performance without collar and its longitudinal application, using the face-only prediction on the young marmosets at 11 months and 16 months (Video 7, Video 7—video supplement 1, Video 7—video supplement 2). The face classifier correctly identified the young twin marmosets solely based on their facial features, indicating that facial identity classification was performed independently of collar information and that the collar beads acted only as an auxiliary cue rather than the main classifier of the system (Video 7).”

The identification program was shown to correctly identify the marmosets; however, we found that “While comparing between the adult and young marmoset datasets, we found that the adult marmosets’ face classifier, trained with a larger number of varied images, showed more reliability and efficiency in marmoset identity recognition.”

The mislabeling was more frequent with the young twins not only due to their face similarity, but also due to the limited number of images being used for model training. The face images used in the identification model training were less compared to the adult model, which contributed to a less accurate prediction result. As the marmoset is still developing before adulthood, their face features will become more different as they age. By increasing the number of training images from different ages of the young marmosets, this could be solved as it is therefore possible to build efficient identification program for longitudinal study. Thus, instead of the current classifiers presented in this manuscript, we suggested that the method/tool could be beneficial for longitudinal studies, not restricting to the individual-based identification program mentioned in this manuscript, as “The tool’s advantages are particularly efficient for large datasets and longitudinal studies, where manual identity labeling becomes difficult, as variability and errors increase along with dataset size and experimenter number.”

(3) How does this method compare to other methods that were used in the past?

The advantages were mentioned in paragraph #1 of the Introduction and paragraph #1-3 in the Discussion. Current approaches for marmosets are usually visible markers (ear dye, collar, etc.), RFID, or observation, of which manual works and human interventions are required. These methods usually need continuous adjustment due to tighter collar, dye fading, etc. This can affect marmoset behaviors, especially during their behavioral task performance, as mentioned as follows:

“While existing marmoset identification approaches usually utilize visible markers, Radio Frequency Identification (RFID), or observation, the manual works and human interventions involved can impact animal behaviors, especially during their behavioral task performance. The facial identification tool aims to collect data from marmosets without having experimenters to check the identity continuously, instead of outperforming the experimenters’ role.”

(4) Did the authors think of adding a continuity or a space constraint? For example, video 6 shows misidentification of the twins; in this specific case, adding a probabilistic continuity or space constraint that will limit identity switches might be useful. This can also be using a retroactive correction - for example, video 3.

We would like to thank the reviewer for this suggestion. We agree that these approaches will be valuable improvements for future offline analysis.

The probabilistic continuity constraint can indeed help decrease identity switches. In our current application, we have implemented a temporal smoothing through majority voting across a 30-frame (1 second) window, of which the program outputs the most frequent prediction of identity. With this strategy, we could reduce the occasional frame misprediction and maintain the real-time performance. Our animals are free-moving and may appear in any location within the camera field of view and housing cage. Therefore, position is not strongly associated with the identity of individual.

We agree that retroactive correction could improve the detection consistency for offline analysis by correcting past detection by future prediction results. However, the current pipeline is incorporated with behavioral tasks, meaning that the prediction results aim to be transmitted with minimal time delay. As additional frame analysis and extra computational power may be needed for retroactive correction, the increased latency can be limited to the utility of real-time system and task control.

Minor issues:

(5) In its current form, I think the manuscript can be significantly shortened and the results/figures can be consolidated (confusion matrices with validation figures for example).

We have followed the reviewer’s suggestion and shortened the Methods and Results sections.

(6) The term "unseen" that the authors use in their results is confusing. Are the authors referring to monkeys that are hidden from their view, or "unseen" before by the model? The video indicates the latter, but I think the term can be changed to something less confusing, like novel, new, etc.

We have changed the term “unseen” by “new” to avoid confusion.

(7) Can the authors add information about the relationship between the number of manually labeled images and the identification precision?

The relationship between number of manually labeled images and identification precision has been described in the Discussion section as: “Moreover, the performance of the system is strongly dependent on the amount and variability of the training data, with identity classification improving as more marmoset images are involved in the model training.”

This means that more manually labeled images (i.e. larger training datasets) could improve the identification precision. However, model performance will plateau regardless of training dataset size, referred in the Results: “Each of the models was trained until reaching the early stopping criteria (i.e. no improvement within the last 100 training epochs).”

More manually labeled images could help improve the variability of model prediction, but too many of these images are also risky for overfitting. In this case, overfitted model might not be able to make valid predictions on new videos/images.

(8) Can the authors expand a bit about the difference between YOLO Nano, small, and medium in the methods?

Thank you for the suggestion. We have added a brief description of the pre-trained models in the methods: “These pre-trained models share the same object detection backbone but differ in number of parameters and computing power. Larger models, such as YOLOv8 medium, provide higher detection accuracy but require greater computational resources and longer inference time. In contrast, smaller models prioritize the computational efficiency.”

(9) Clear and short definitions of what IoU, Recall, F1, and other terms represent should be added to the results section (not formulas, short sentences).

The definitions and formula for the evaluation metrics were described in detail in the Methods section. To help readers while avoiding repetitions with the detailed methodology definitions, we have added a brief description of these terms at their first mention in the Results section: “Model performance included the precision (the proportion of correct positive predictions), recall (the proportion of corrected predicted ground-truth labels), and mAP@50–95 (the average detection accuracy across different IoU object localization thresholds; see Methods and Materials section for detailed definitions).”.

(10) In the methods-"video collection" section, can the authors please include more information? Is this a motion-sensitive camera? Otherwise, what's the size of the data that is collected? This will help in reproducibility and system requirements. If this is not continuously collected data, discuss what can be done to make an online identification tool.

We have included the camera as industrial color/RGB camera, which functions like any webcam and has no motion-sensitive functions. The size of data collected was described in detail in the Methods section that we slightly modified for clarity for: “Three adult marmosets from one family were recorded for 1 hour across each of the 5 recording days, with unrestricted voluntary access to the primate chair space. For the adult marmosets, the housing cage door was opened at the beginning of the recording session allowing them to enter and exit freely into the primate chair space for food rewards and observation (Figure 1C). Two young marmosets were briefly isolated and recorded separately for testing and improving the automatic face extraction program. We recorded them at two developmental time points, 7 months old and 11 months old (an additional time point at 16 months old has been added for one marmoset to test the identification without collar). During video collection, a sliding panel and an in-cage box were positioned near the housing cage door to temporarily isolate individual marmosets from other family members. Individual isolation was kept brief (approximately 10 minutes) to prevent disturbance and potential stress due to family separation. “To capture sufficient variability in postures, individuals, and lighting conditions, clips were sampled throughout the adult marmoset videos (approximately 5 hours) (Figure 2B).”

The size of the training dataset was also described in detail in the Methods section, referred as: “To minimize image computations and data storage, we created a dataset of 2498 annotated images from the three adult marmosets. All images were manually annotated to label marmoset faces, individual identities, and the collar bead colors (Figure 2C). The annotated images were used for training models of multi-marmoset face classification and the automatic identity extraction, which can automatically detect, localize, and identify marmoset faces (Figure 2A, Step 1 – 4). We created another dataset of two young marmosets at 7 months old (total images = 502) for testing the automatic facial and identity extraction (Figure 2A, Step 4 – 5). For both adult and young marmoset datasets, images were randomly divided into a training set and a validation set at a ratio of 8:2.”

Our manuscript is not describing an online identification tool (i.e. the described program does not require connection to internet). Instead, once trained and the program is performing well with new marmoset videos, we could use the trained weights for real-time marmoset identification (no need to collect new training data) as we are doing it for our touchscreen data collection in cage. It is referred in the Discussion as follows: “Once trained, the system operates automatically to collect real-time identity and can work to present subject-specific behavioral or cognitive tasks based on the identity of the detected animal, with no work or presence needed on the user’s end. This tool has already been implemented in touchscreen-based marmoset cognitive tasks, including pairwise visual discrimination paradigm.”

This program can be used for online applications if you are using a camera that is connected 24/7. For now, we are only using it while using our behavioral testing chair due to limitations issues (safety recordings, removing all electrical apparatus during night, overheating of camera if use continuously).

(11) Would increasing the size of the beads help with their identification? In the images included in Figure 2C, it's very difficult to see these beads.

Yes, collar beads can be occluded by fur, blurred by motions, or outside the field of camera view. We have now included increasing the collar beads size in the Discussion, referred as: “An alternate experimental solution is to improve collar visibility, including using distinct color code across individuals within a family, increasing the size of the beads, or increasing collar beads number to reduce occlusion.” However, the size of the beads needs to be appropriate to avoid being inconvenient and disruptive to the animals to ensure their welfare.

(12) It's difficult to understand the setup of the camera in regard to the housing cage. Can the marmosets go into the primate's chair at any point (from the videos, it seems so, but the dashed line in 1C might indicate otherwise), or is the primate chair there only to mount the camera? Consider redrawing 1C in a more clear way.

The figure 1C is a simplified drawing of the photo 1A, we have edited the drawing of Figure 1C to highlight the free access when the chair is mounted to the cage as stated here: “For the adult marmosets, the housing cage door was opened at the beginning of the recording session allowing them to enter and exit freely into the primate chair space for food rewards and observation (Figure 1C).”

(13) Given the repetitiveness of the figures, an icon atop each figure specifying what's being tested will be helpful.

We have added an icon for each figure 3-8.

(14) I feel the supplemental figures for Figure 9 are more compelling than the main figure. Consider including some of the panels in the main figure.

Thank you for the suggestion. We included heat maps of the adult family relationships in Figure 9, including the cosine similarities and Euclidean distances. The current legend of Figure 9 is changed as follows: “Figures 9. Across-model visualization of the face similarity between marmoset pairs. Four types of family relationships (mother-father, father-son, mother-son, and twin1-twin2) were compared, based on the training results of adult and young marmosets. The similarity was calculated using (A) cosine similarity and the (B) Euclidean distance. Heat map of the (C) cosine similarity and (D) Euclidean distance was plotted between the 3 relationship pairs in the adult family. The cosine similarity score ranged from 0 (very different) and 1 (exactly same) for marmoset faces. The Euclidean distance score ranged from 0 (exactly same) and 1 (very different) for marmoset faces.”

(15) Related to this, I feel that the display of Figure 9 obscures the differences that the authors report.

The display of Figure 9 has been improved following the above reviewer’s suggestions.

(16) In Figure 9, given the large effect sizes but non-significant p-values, will adding more training points/epochs improve the differences.

For the trained recognition programs, we have already implemented the “early stopping criteria” as shown in the Results section: “Each of the models was trained until reaching the early stopping criteria (i.e. no improvement within the last 100 training epochs).” This means that the program has already plateau with its performance.

(17) Line 30: either "within" or "in".

This has been corrected.
